# Synthesis of New Azo Compounds Based on *N*-(4-Hydroxypheneyl)maleimide and *N*-(4-Methylpheneyl)maleimide

**DOI:** 10.3390/molecules15107498

**Published:** 2010-10-25

**Authors:** Issam Ahmed Mohammed, Asniza Mustapha

**Affiliations:** School of Industrial Technology, Universiti Sains Malaysia, 11800 Penang, Malaysia

**Keywords:** synthesis, azo compounds, aromatic amines, N-(4-hydroxylpheneyl)maleimide

## Abstract

Maleic anhydride was reacted with *p*-aminophenol and *p*-toluidine in the presence of di-phosphorus pentoxide (P_2_O_5_) as a catalyst to produce two compounds: *N*-(4-hydroxy-phenyl)maleimide (**I**) and *N*-(4-methylphenyl)maleimide (**II**). The new azo compounds **I(a-c) **and **II(a-c) **were prepared by the reaction of **I **and **II** with three different aromatic amines, namely aniline, *p*-aminophenol and *p*-toluidine. The structures of these compounds were confirmed by CHN, FT-IR, ^1^H-NMR, ^13^C-NMR, mass spectrum and UV/Vis spectroscopy.

## 1. Introduction

Small molecules and macromolecules containing imide groups exhibit great electrical properties, good solubility in polar media, resistance to hydrolysis and high thermal stability [1,2,3,4,5,6,7,8]. Due to their excellent properties many efforts have been made to produce different compounds containing imide groups consisting of two carbonyl groups bound to nitrogen. The most common unsubstituted cyclic imides were prepared by heating dicarboxylic acids or their anhydrides with reactants including ammonia, urea, formamide lithium nitride or primary amines [9,10,11,12], but the reaction needs to be carried out at high temperatures for efficient ring closure. Recently, attempts at preparing imide compounds either by the conventional technique or via the microwave irradiation using various catalysts such as Lewis acids, hexamethyldisilazane, carbonyldiimidazole, 4-*N,N*-dimethylaminopyridine, ammonium chloride, hydroxylamine hydrochloride and sodium acetate to minimize the temperature and time of the reaction have been published [13,14,15,16,17,18]. In this study, the conventional technique was used to synthesize two imides by the reaction of maleic anhydride with *p*-aminophenol and *p*-toluidine, respectively, in the presence of diphosphorus pentoxide (P_2_O_5_) as a catalyst, which decreased the temperature needed for ring closure from 150–300 °C to 20–70 °C.

## 2. Results and Discussion

### 2.1. Synthesis and characterization

**Scheme 1:**
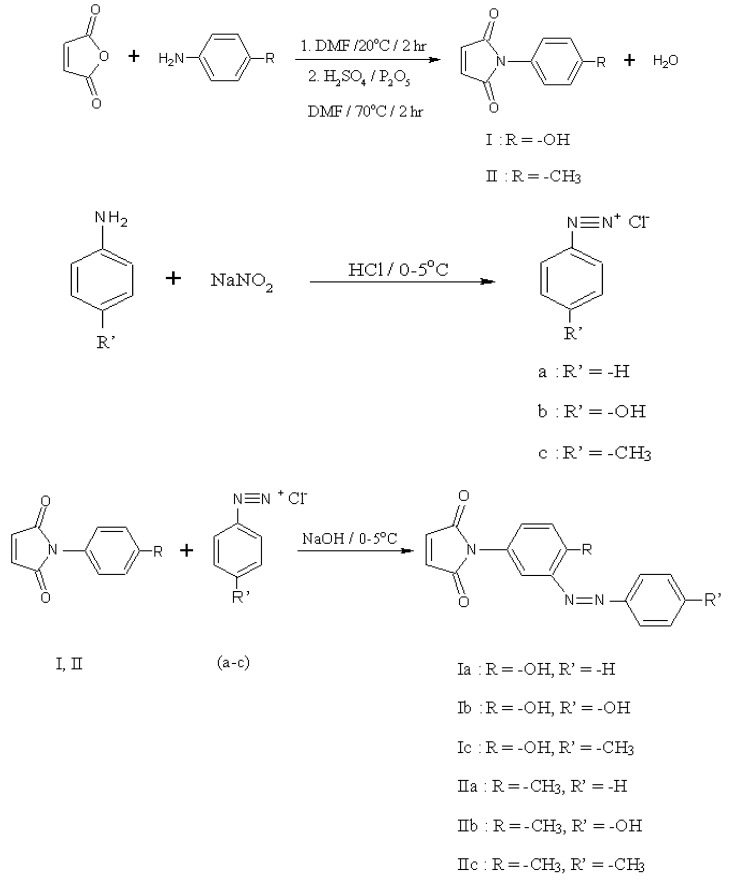
Synthesis of N-(4-hydroxypheneyl)maleimide (**I**), N-(4-methylpheneyl)maleimide (**II**), **I(a-c) **and** II(a-c)**.

The preparation of compounds **I**, **II**, **I(a-c)** and **II(a-c)** is shown in Scheme 1. The structure of these compounds was confirmed by elemental analysis (CHN), FT-IR, ^1^H-NMR, ^13^C-NMR, mass spectrum and UV/Vis spectroscopy. 

The FT-IR spectra of compounds **I** and **II** showed the presence of C=O absorbances at 1,702 cm^−1^, alkene group (HC=CH) ones at 3,119 cm^−1^ and the presence of aromatic rings indicated by bands at 1,589, 1,600 and 1,512 cm^−1^. In addition a hydroxyl group at 3,481 cm^−1^ and a methyl group at 1,316 cm^−1^ were seen for compounds **I** and **II**, respectively.

**Figure 1:**
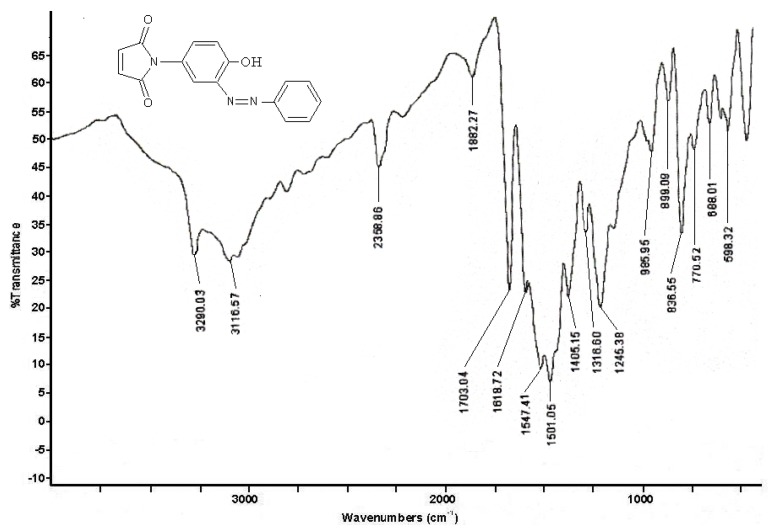
FT-IR spectrum of compound **Ia**.

All these peaks clearly proved that compounds **I** and **II **were produced. The FT-IR data for compounds **I(a-c)** and **II(a-c)** showed the same characteristic bands of the coupling agents **I** and **II**, namely imide, methyl, hydroxyl group, alkene, and *p*-substituted band while the presence of the azo (N=N) group band in 1,630–1,575 cm^−1^ range confirmed the success of the synthesis. Besides, the *o*-substituted benzene ring absorbtion at 750–775 cm^−1 ^proved that the azo group was attached to the *ortho* position of the benzene rings. The FT-IR spectrum of compound **Ia** as a typical example is shown in Figure 1.

**Figure 2:**
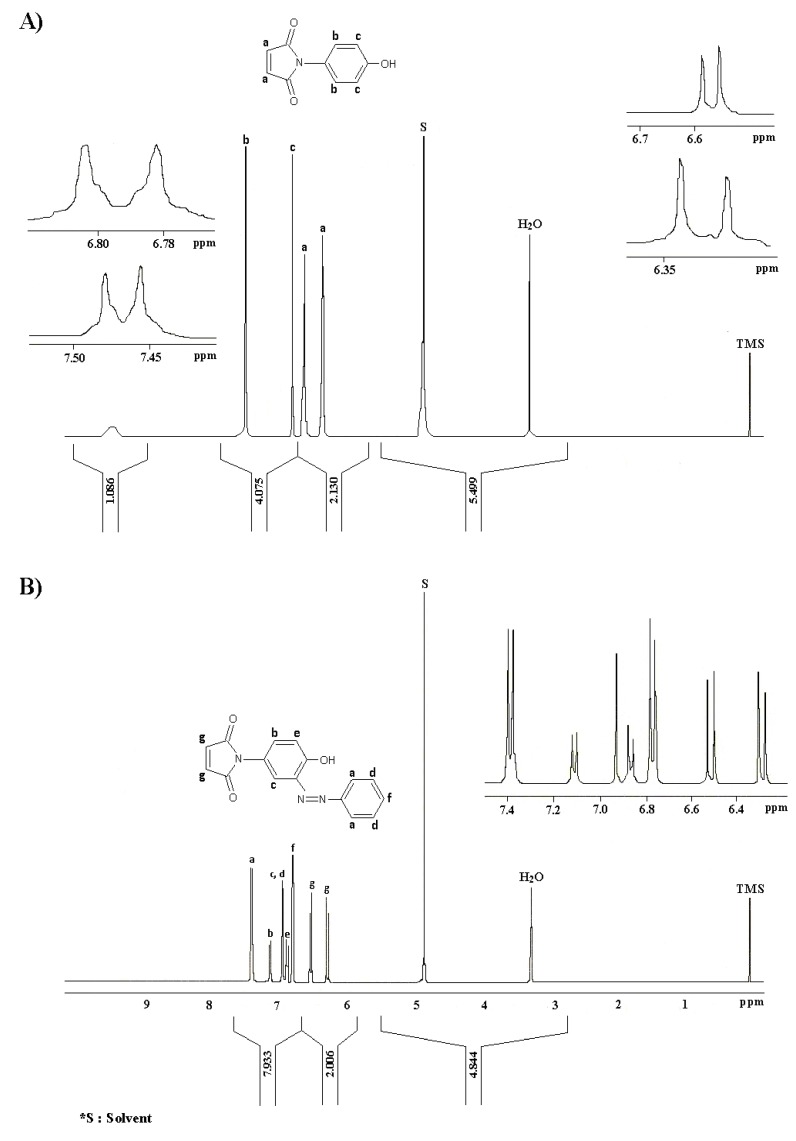
^1^H-NMR spectra of (A) compound **I** and (B) compound **Ia **in CD_3_OD.

**Figure 3:**
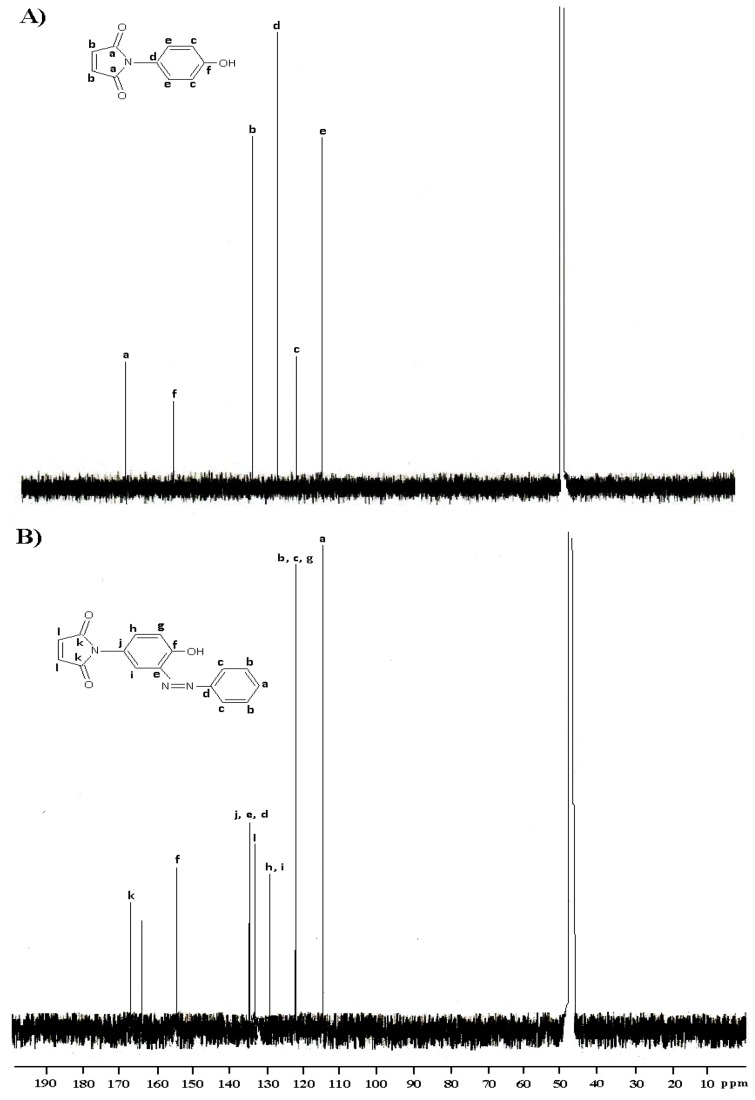
^13^C-NMR spectra of (A) compound **I** and (B) compound **Ia **in CD_3_OD.

The ^1^H-NMR and ^13^C-NMR for azo compounds **I** and **Ia** have been chosen as typical examples and the corresponding spectra are shown in Figure 2 and Figure 3, respectively. In the ^1^H-NMR spectrum, the protons of the alkene group (HC=CH) and the protons of aromatic ring appeared at 6.62–6.52 ppm and 6.75–7.39 ppm, respectively. The new peak appeared at 6.93 ppm was assigned to the *ortho* position and that proved the reaction between compound **I** and aniline has occurred. The broad peak at 9.45–9.75 ppm was assigned to the free O-H proton. 

In the ^13^C-NMR spectrum, the following signals are the characteristic of the structure; 167.43 ppm (C=O), 155.44 ppm (C-O, aromatic), 134.87 ppm (HC=CH, alkene) and 122.56, 115.42 ppm (C=C, aromatic). 

**Figure 4:**
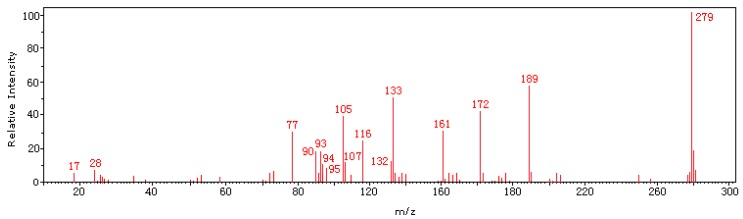
Mass spectrum of compound **Ia**.

The addition of other aromatic carbons proved that the pure azo compound **Ia** has been successfully prepared. The mass spectrum (70 eV) of **Ia** (Figure 4) shows the presence of molecular ion peak as the base peak at m/z 279 (100). The major fragmentation of molecular ion occurs through the loss of phenyldiazonium at m/z 189 (58.0). On further fragmentation it gives peaks at m/z 172 (42.56), 161 (32.5), 133 (12.05), 132 (12.0), 116 (27.0), 107 (11.05), 105 (8.7), 94 (10.59), 93 (17.82), 90 (18.0), 77 (31.04). 

In the UV/Visible spectra, the azo group (N=N) usually gives an absorption in the 350–370 nm range [19]. We expected compounds **I(a-c)** would exhibited higher λ_max_ than compounds **II(a-c)** due to the presence of an auxochrome group such as hydroxyl group in the compounds [20]. However, the results showed the opposite, whereby they gave a lower absorption wavelength, which is in the range of 320–330 nm. This might be attributed to the tautomerism due to the polar or proton donor solvents, which can stabilize the carbonyl group by dipolar association or hydrogen bonding and thus decrease the magnitude of the enolization. As the result, the keto group will give a shorter wavelength [21]. The appearance of a new peak at 164 cm^−1 ^in the ^13^C-NMR spectrum (Figure 3) suggests that this phenomenon could have indeed occurred. The UV/Vis data for the azo compounds **I(a-c)** and **II(a-c)** are given in the Experimental section.

## 3. Experimental

### 3.1. Materials

Maleic anhydride (R&M Chemicals, UK), *p*-aminophenol (Sigma-Aldrich, UK), aniline (Fisher Chemicals, UK), *p*-toluidine (Fluka, Germany), sulfuric acid 98% (Mallinckrodt, Mexico), hydrochloric acid 37% (Fisher Chemicals, UK), sodium hydroxide (R&M Chemicals, UK), *N,N*-dimethylformamide (Systerm®, Malaysia), diphosphorus pentoxide (Scharlau Chemie, Spain), sodium nitrite (Ajax Chemicals, Australia), 2-propanol (R&M Chemicals, UK) and glacial acetic acid (Fisher Chemicals, UK). All the chemicals were used as received without further purification except for aniline, was distilled before use.

### 3.2. Instrumentation

FT-IR spectra were measured at room temperature using a Perkin-Elmer 2000 FT-IR equipped with a high-purity dried potassium bromide (KBr) beam splitter. The ^1^H-NMR and ^13^C-NMR spectra were obtained using a Bruker 400 MHz NMR spectrophotometer with tetramethylsilane (TMS) as the internal reference. CHN microanalyses were performed using Perkin Elmer 2400 Series II Combustion Analyzer. The MS were recorded on a Perkin Elmer Clarus 500 Gas Chromatography-Mass Spectrometry system (GC-MS). UV/Vis Spectroscopy were determined using a Shimadzu UV-1601 PC instrument. The entire sample was weighed and dissolved in methanol.

### 3.3. Synthesis of N-(4-hydroxyphenyl)maleimide *(**I**)*

*P*-aminophenol (16.37 g, 0.15 mol) and maleic anhydride (14.71 g, 0.15 mol) were dissolved separately in DMF (50 mL) to yield solutions A and B, respectively. Solution B was added dropwise into solution A to give solution C. Solution C was stirred for 2 hours at 20 °C in a water bath. P_2_O_5_ (12 g) was dissolved in H_2_SO_4_ (10 mL) and DMF (70 mL). This mixture was added dropwise into solution C and was stirred for 2 hours at 70 °C. The mixture was kept chilled in the ice bath and poured into cold water. A precipitate formed that was filtered, washed with distilled water and finally recrystallized from 2-propanol and dried in a vacuum oven at 65 °C for 24 hours. Yield was 84%; m.p. 182–184 °C; color: yellow; FT-IR (KBr disc): 3,481 cm^−1 ^(O-H), 3,108 cm^−1 ^(HC=HC), 1,705 cm^−1 ^(C=O), 1,601 cm^−1 ^(aromatic ring) and 828 cm^−1^ (HC=CH of maleimide); ^1^H-NMR (CD_3_OD): 6.78–7.48 (aromatic), 6.90–7.15 (HC=CH of maleimide), 6.31–6.58 (HC=CH) ppm; ^13^C-NMR (CD_3_OD): 170.79 (C=O), 157.44, 134.37 (HC=CH of maleimide), 128.23, 112.00 (C=C, aromatic) ppm.

### 3.4. Synthesis of N-(4-methylphenyl)maleimide *(**II**)*

Compound** II** was prepared by following the procedure of the preparation of **I** except that *p*-toluidine was substituted for *p*-aminophenol. Yield was 50% with a melting point of 148–150 °C; color: yellow; FT-IR (KBr disc): 3,088 cm^−1 ^(HC=CH), 1,708 cm^−1 ^(C=O), 1,632 cm^−1 ^(aromatic ring), 1,316 cm^−1^ (CH_3_) and 823 cm^−1 ^(*p*-substituted Ar); ^1^H-NMR (CD_3_OD): 7.17–7.57 (aromatic), 6.98–7.21 (HC=CH of maleimide), 6.32–6.55 (HC=CH), 2.35 (CH_3_) ppm; ^13^C-NMR (CD_3_OD): 164.99 (C=O), 134.48 (HC=CH of maleimide), 128.11, 126.36, 125.81, 120.71 (C=C, aromatic), 19.96 (CH_3_) ppm.

### 3.5. General procedure for preparation of the heterocyclic azo compounds ***I(a-c)*** and ***II(a-c)***

Solution A was prepared by mixing pure aniline (**a**, 0.93 g, 0.01 mol) with concentrated HCl (3 mL) and water (3 mL) and cooling at 5 °C in an ice bath. NaNO_2_ (0.69 g, 0.01 mol) was dissolved in water (10 mL) at 5 °C to obtain solution B. Then solution A was added dropwise to solution B at 5 °C with stirring. The mixture was then slowly added into the solution of compound **I** (1.89 g, 0.01 mol), which was dissolved in 10% NaOH (20 mL) at 5 °C. The mixture was keep chilled in the ice bath and stirred continuously for 10 min. The precipitate formed was filtered and recrystallized from glacial acetic acid, and washed with methanol and finally dried in a vacuum oven at 65 °C for 24 hours. The procedure was repeated by substituted **I** with **II**, where was substituted by *p*-aminophenol (**b**) and *p*-toluidine (**c**)*.*

*Phenylazo-3-N-(4-hydroxyphenyl)maleimide* (**Ia**). Color: pale yellow; yield: 85%; melting point: 199–200 °C; FT-IR (KBr disc): 3,290 cm^−1 ^(O-H), 3,116 cm^−1 ^(HC=CH), 1,703 cm^−1 ^(C=O), 1,618 cm^−1 ^(aromatic ring), 1,547 cm^−1^ (N=N), 836 cm^−1^ and 770 cm^−1^; ^1^H-NMR (CD_3_OD): 7.20–7.43 (aromatic), 6.93–7.25 (HC=CH of maleimide), 6.26–6.52 (HC=CH) ppm; ^13^C-NMR (CD_3_OD): 167.43 (C=O), 165.01 (C=O), 155.44, 134.87 (HC=CH of malemide), 122.56, 115.42 (C=C, aromatic) ppm; elemental analysis: found: C, 62.76; H, 7.65; N, 13.86, (C_16_H_23_N_3_O_3_), calc.: C, 62.92; H, 7.60; N, 13.77; UV/Vis λ_max _(nm): 327.00 (N=N); 302.50 (C=O); 230.00 (Ar-CN); 208.50 (Ar-OH).

*4-Hydroxyphenylazo-3-N-(4-hydroxyphenyl)maleimide* (**Ib**). Color: yellow; yield: 81%; melting point: 210–212 °C; FT-IR (KBr disc): 3,302 cm^−1 ^(O-H), 3,166 cm^−1 ^(HC=CH), 1,701 cm^−1 ^(C=O), 1,618 cm^−1 ^(aromatic ring) and 1,580 cm^−1^ (N=N), 835 cm^−1 ^and 716 cm^−1^; ^1^H-NMR (CD_3_OD): 7.17–7.57 (aromatic), 6.96–7.21 (HC=CH of maleimide), 6.30–6.51 (HC=CH) ppm; ^13^C-NMR (CD_3_OD): 170.95 (C=O), 155.71, 155.36, 134.77 (HC=CH of maleimide), 129.93, 122.84, 112.00 (C=C, aromatic) ppm; elemental analysis: found: C, 60.12; H, 6.98; N, 13.10, (C_16_H_23_N_3_O_4_), calc.: C, 60.01; H, 6.93; N, 13.17; UV/Vis λ_max _(nm): 326.50 (N=N); 302.50 (C=O); 230.50 (Ar-CN); 210.00 (Ar-OH).

*4-Methylphenylazo-3-N-(4-hydroxyphenyl)maleimide* (**Ic**). Color: yellow; yield: 80%; melting point: 203–204 °C; FT-IR (KBr disc): 3,302 cm^−1 ^(O-H), 3,202 cm^−1 ^(HC=CH), 1,703 cm^−1 ^(C=O), 1,618 cm^−1 ^(aromatic ring), 1,511 cm^−1^ (N=N), 835 cm^−1^ and 719 cm^−1^; ^1^H-NMR (CD_3_OD): 7.17–7.52 (aromatic), 6.90–7.20 (HC=CH of maleimide), 6.29–6.53 (HC=CH), 2.35 (CH_3_) ppm; ^13^C-NMR (CD_3_OD): 170.11 (C=O), 155.20, 134.48 (HC=CH of maleimide), 133.66, 129.74, 127.00, 125.00, 122.76, 112.00 (C=C, aromatic), 20.01 (CH_3_) ppm; elemental analysis: found: C, 64.09; H, 7.84; N, 13.18, (C_17_H_25_N_3_O_3_), calc.: C, 63.91; H, 7.89; N, 13.16; UV/Vis λ_max _(nm): 330.00 (N=N); 302.50 (C=O); 231.50 (Ar-CN); 210.00 (Ar-CH_3_).

*Phenylazo-3-N-(4-methylphenyl)maleimide* (**IIa**). Color: yellow; yield: 82%; melting point: 185–186 °C; FT-IR (KBr disc): 3,092 cm^−1 ^(HC=CH), 1,703 cm^−1 ^(C=O), 1,630 cm^−1 ^(aromatic ring), 1,540 cm^−1^ (N=N), 758 cm^−1^ and 1,314 cm^−1^ (CH_3_); ^1^H-NMR (CD_3_OD): 7.17–7.53 (aromatic), 6.96–7.19 (HC=CH of maleimide), 6.32–6.59 (HC=CH), 2.35 (CH_3_) ppm; ^13^C-NMR (CD_3_OD): 164.99 (C=O), 134.75 (HC=CH of maleimide), 135.29, 133.55, 129.43, 128.11, 126.36, 125.81, 120.71 (C=C, aromatic), 19.96 (CH_3_) ppm; elemental analysis: found: C, 67.46; H, 8.14; N, 13.91, (C_17_H_25_N_3_O_2_), calc.: C, 67.28; H, 8.31; N, 13.86; UV/Vis λ_max _(nm): 340.50 (N=N); 225.50 (Ar-CN); 205.50 (phenyl).

*4-Hydroxyphenylazo-3-N-(4-methylphenyl)maleimide* (**IIb**). Color: yellow; yield: 83%; melting point: 190–191 °C; FT-IR (KBr disc): 3,210 cm^−1 ^(O-H), 3,093 cm^−1 ^(HC=CH), 1,703 cm^−1 ^(C=O), 1,630 cm−^1 ^(aromatic ring), 1,541 cm^−1^ (N=N), 758 cm^−1^ and 1,312 cm^−1^ (CH_3_); ^1^H-NMR (CD_3_OD): 7.19–7.57 (aromatic), 6.95–7.20 (HC=CH of maleimide), 6.32–6.48 (HC=CH), 2.35 and 2.40 (CH_3_) ppm; ^13^C-NMR (CD_3_OD): 167.12 (C=O), 155.68, 143.19, 136.46, 136.01, 134.78 (HC=CH of maleimide), 129.11, 128.26, 127.43, 112.01 (C=C, aromatic), 19.93 (CH_3_) ppm; elemental analysis: found: C, 63.83; H, 7.89; N, 13.12, (C_17_H_25_N_3_O_3_), calc.: C, 63.91; H, 7.89; N, 13.16; UV/Vis λ_max _(nm): 366.50 (N=N); 225.50 (Ar-CN); 207.00 (Ar-OH).

*4-Methylphenylazo-3-N-(4-methylphenyl)maleimide* (**IIc**). Color: pale yellow; yield: 85%; melting point: 186–187 °C; FT-IR (KBr disc): 3,091 cm^−1 ^(HC=CH), 1,703 cm^−1 ^(C=O), 1,630 cm^−1 ^(aromatic ring), 1,540 cm^−1^ (N=N), 778 cm^−1 ^and 1,315 cm^−1^ (CH_3_); ^1^H-NMR (CD_3_OD): 7.20–7.59 (aromatic), 6.90–7.19 (HC=CH of maleimide), 6.32–6.55 (HC=CH), 2.39 (CH_3_) ppm; ^13^C-NMR (CD_3_OD): 165.36 (C=O), 144.33, 134.80 (HC=CH of maleimide), 128.08, 127.11, 126.47, 112.33 (C=C, aromatic), 20.10, 20.03, 19.96 (CH_3_) ppm; elemental analysis: found: C, 68.01; H, 8.72; N, 13.19, (C_18_H_27_N_3_O_2_); calc.: C, 68.14; H, 8.58; N, 13.24; UV/Vis λ_max _(nm): 352.00 (N=N); 225.50 (Ar-CN); 206.00 (Ar-CH_3_).

## 4. Conclusions

Six new azo compounds based on N-(4-hydroxyphenyl)maleimide and N-(4-methylphenyl)maleimide have been successfully synthesized and characterized. The use of P_2_O_5_ as catalyst has minimized the reaction temperature from 150–300 °C to 20–70 °C as well as giving high yields.
